# Effects of Direct Social Experience on Trust Decisions and Neural Reward Circuitry

**DOI:** 10.3389/fnins.2012.00148

**Published:** 2012-10-16

**Authors:** Dominic S. Fareri, Luke J. Chang, Mauricio R. Delgado

**Affiliations:** ^1^Department of Psychology, Rutgers UniversityNewark, NJ, USA; ^2^Department of Psychology and Neuroscience, University of ColoradoBoulder, CO, USA; ^3^Department of Psychology, University of ArizonaTucson, AZ, USA

**Keywords:** trust, learning, social experience, reward, striatum, prediction error

## Abstract

The human striatum is integral for reward-processing and supports learning by linking experienced outcomes with prior expectations. Recent endeavors implicate the striatum in processing outcomes of social interactions, such as social approval/rejection, as well as in learning reputations of others. Interestingly, social impressions often influence our behavior with others during interactions. Information about an interaction partner’s moral character acquired from biographical information hinders updating of expectations after interactions via top down modulation of reward circuitry. An outstanding question is whether initial impressions formed through experience similarly modulate the ability to update social impressions at the behavioral and neural level. We investigated the role of experienced social information on trust behavior and reward-related BOLD activity. Participants played a computerized ball-tossing game with three fictional partners manipulated to be perceived as good, bad, or neutral. Participants then played an iterated trust game as investors with these same partners while undergoing fMRI. Unbeknownst to participants, partner behavior in the trust game was random and unrelated to their ball-tossing behavior. Participants’ trust decisions were influenced by their prior experience in the ball-tossing game, investing less often with the bad partner compared to the good and neutral. Reinforcement learning models revealed that participants were more sensitive to updating their beliefs about good and bad partners when experiencing outcomes consistent with initial experience. Increased striatal and anterior cingulate BOLD activity for positive versus negative trust game outcomes emerged, which further correlated with model-derived prediction error learning signals. These results suggest that initial impressions formed from direct social experience can be continually shaped by consistent information through reward learning mechanisms.

## Introduction

Social interactions are governed by social norms and expectations that enable us to learn the reputation of others in order to establish relationships (Fehr and Camerer, 2007; Rilling and Sanfey, 2011). One factor critical to the development of meaningful social relationships is trust. Trust is predicated on a notion of reciprocity, that generous or kind behavior toward another will be reciprocated (Berg et al., 1995; van den Bos et al., 2009). This expectation innervates relationships across all facets of our social lives – business partnerships, friendships, romantic relationships. Though crude appraisals of trustworthiness can be made rapidly (Adolphs et al., 1998; Willis and Todorov, 2006; Engell et al., 2007; Todorov et al., 2008), decisions to pursue any kind of social relationship often require learning one’s reputation via experience through repeated interactions, akin to trial and error learning (Chang et al., 2010). These experiences can be colored by prior social expectations such as implicit racial attitudes (Stanley et al., 2011), or knowledge of moral character (Delgado et al., 2005a), which can influence decisions to trust and neural processing of social outcomes. It is unclear, however, whether social impressions acquired through direct social experience in a different domain can generalize and bias trust behavior, as measured by performance in an economic game, and associated neural mechanisms involved in reputation building.

An extensive body of work has delineated a putative neural circuitry involved in reward-processing (for review see Haber and Knutson, 2010). Of particular interest, the literature highlights a role for the striatum and regions of medial prefrontal cortex (mPFC) in coding reward outcome value (Robbins and Everitt, 1996; Delgado et al., 2000, 2003; Rolls, 2000; Knutson et al., 2001; O’Doherty et al., 2002; Haber and Knutson, 2010). The human striatum has been posited to be involved in learning through establishing links between actions and experienced outcomes (Tricomi et al., 2004; Delgado et al., 2005b; Galvan et al., 2005); the striatum further receives modulatory input from midbrain dopaminergic (DA) nuclei thought to compute prediction error (PE) learning signals (i.e., the difference between expected and received reward; Schultz et al., 1997; Hollerman and Schultz, 1998; Sutton and Barto, 1998; Niv and Schoenbaum, 2008). Findings from human neuroimaging studies demonstrate correlates of PE signaling in the striatum (O’Doherty et al., 2003, 2004; Pessiglione et al., 2006; Schonberg et al., 2007; Daw et al., 2011; Li et al., 2011b).

Equally important is the notion that valuation and learning based on social outcomes (e.g., social approval, positive emotional responses, peer feedback) seem to be coded similarly in reward circuitry (Izuma et al., 2010; Jones et al., 2011; Lin et al., 2012). This extends to even more abstract social rewards such as reciprocity, which can provide information about one’s reputation and guide behavior in repeated interactions. For example, increased striatal BOLD responses emerge when trustees in a repeated trust game experience positive reciprocity from investors (e.g., investments are better than expected), and these BOLD responses propagate backward in time to before revelation of the investors’ decisions as reputation for reciprocity is learned (King-Casas et al., 2005). This pattern mirrors that observed in recordings of midbrain DA neurons which fire to the earliest predictors of positive outcomes (Schultz et al., 1997), implicating similar mechanisms at play during social learning (Kishida and Montague, 2012). Subsequent work has converged on the notion that perceiving trustworthiness and learning reputation develops dynamically over time as a function of changing social situations (Chang et al., 2010) and partner reciprocity (van den Bos et al., 2009). These processes are highly dependent upon corticostriatal reward circuitry, particularly the ventral striatum and mPFC (Krueger et al., 2007; Phan et al., 2010).

The ability to learn a partner’s reputation can be highly sensitive to previously formed impressions of others. For example, perceptions of moral character attained via instructed or third party means prior to social interactions modulate both subsequent decisions to trust others, as well as the ability of neural reward circuitry to effectively process outcomes from trust interactions (Delgado et al., 2005a). In said study, participants learned about the moral aptitude of three fictional partners via instructed means (e.g., reading a biographical vignette), forming impressions that the partners were either of praiseworthy, negative or neutral moral character. In a subsequent trust game with these partners, participants served as the investors in repeated trials. Even though the partners played at a fixed 50% reinforcement rate, the initial instructed biases influenced participants’ decision-making such that they did not adapt behavior according to experienced outcomes; this was also reflected by aberrant outcome processing signals in the striatum.

**Figure 1:**
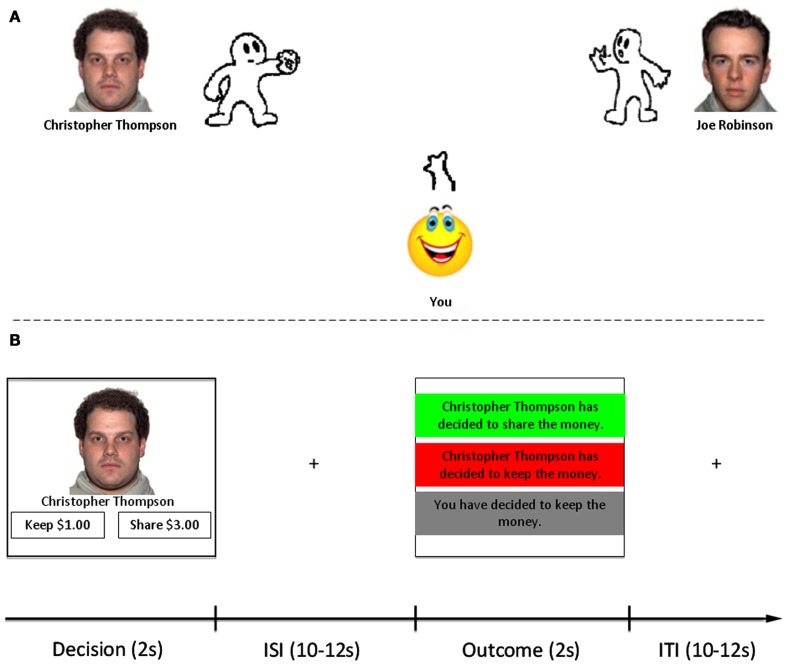
**Task structure**. **(A)** Participants were first introduced to three different partners with whom they would later interact with in the trust game. Participants played three separate versions of Cyberball. The character on the right side of the screen was consistent across all three versions (control). The face and name of the character on the left side changed in each version, as did character ball-tossing behavior to be depicted as good, bad, neutral. **(B)** After Cyberball, participants played an iterated trust game adapted from Delgado et al. (2005a). Trials with each partner were interleaved within functional scanning runs, as were lottery trials (non-social control). Each trial consisted of a Decision Phase (2 s) in which they were to choose whether to keep or share money with their partner (or not play/play the lottery). After a variable ISI (10–12 s), the Outcome Phase (2 s) was presented consisting of feedback in the form of partner reciprocation/defection, or null feedback indicating defection by the participant. All trials were separated by a 10–12 s ITI.

Instructed learning is one method by which we may acquire information about another person’s reputation. We can also have direct experiences with others in separate domains that shape initial impressions before subsequent interactions. For example, we could experience a co-worker as aggressive or bossy in a work environment, and bring this initial impression to subsequent interactions in a more recreational setting. It is unclear whether learning through direct social experience modulates trust behavior similarly to instructed learning. To investigate this, we employed a two-step approach (see Figure 1). We first employed a learning phase in which we manipulated personalities of fictional partners in a computerized ball-tossing game (Cyberball; Williams et al., 2000) to be perceived as either “good,” “bad,” or “neutral.” Participants played this game prior to the second step, in which they interacted with these same partners in a modified economic trust game (Delgado et al., 2005a) while undergoing fMRI. Importantly, all partners were programmed to demonstrate similar reciprocation patterns in the trust game, irrespective of their personalities in Cyberball. We hypothesized that social impressions formed from direct social experience would shape perceptions of trustworthiness, subsequent behavior in the trust game, and neural circuitry supporting reputation building.

## Materials and Methods

### Participants

Twenty-five participants (12 female, mean age = 22.32, SD = 5.58) from Rutgers University-Newark and the surrounding area were recruited for this study using posted advertisements. Imaging sessions were conducted at the University Heights Advanced Imaging Center at the University of Medicine and Dentistry of New Jersey (Newark, NJ, USA). All participants undergoing fMRI were screened for history of psychiatric illness and head trauma, and all provided informed consent before participating. A total of seven participants were excluded from analysis due to: excessive head motion (>3 mm in at least one plane across more than one run of the experiment; two participants); equipment malfunction in the scanner (one participant); failure to comply with task requirements, specifically falling asleep for one or more functional runs (two participants), resulting in an excessive amount of missed trials (>1.5 SD from the mean of the group: mean = 4.5, SD = 6.72); and insufficient variability in behavioral data to allow modeling of imaging and behavioral data (see explanation below; two participants). Behavioral and imaging analyses were conducted on the remaining 18 participants (nine female, mean age = 23.06 years, SD = 6.41). All participants received monetary compensation at a rate of $25/h for their participation in the task. Additional bonuses were paid based on outcomes from randomly chosen trials in the task. The Internal Review Boards of both Rutgers University and the University of Medicine and Dentistry of New Jersey approved this study.

### Experimental paradigm

The experiment took place over a total of 2 days, separated by no more than 1 week. The imaging session took part on Day 1. Participants returned on Day 2 to perform a follow-up behavioral session. Participants provided informed consent and completed an fMRI screening form to ensure requirements for undergoing fMRI were met on Day 1. After completion of these consent and screening forms, participants were instructed and trained on the task.

Participants were told that they would be playing an economic exchange game called the trust game with three fictional partners (facial stimuli taken from the NimStim database, Tottenham et al., 2009) whom they would first encounter through playing a computerized ball-tossing game called Cyberball (Williams et al., 2000). With the aim of mimicking the instructed learning procedures of Delgado et al. (2005a) – three short bios depicting moral character – we administered three short versions of the Cyberball game, each consisting of a total of 20 throws. Participants played with two partners at a time in each game, one on either side of the computer screen (see Figure 1A). One partner (on the right side of the screen) was the same in all three versions of the game (control), and participants were instructed that they would not interact with this partner after Cyberball. The other partner (on the left side of the screen) changed in each version of the game; these were the partners with whom participants would interact with later. Participants were instructed to simply to toss a ball back and forth with the partners on screen. They were not made explicitly aware of the fact that the partners’ personalities were manipulated to foster social impressions: one always threw to the participant, never to the control (good); another never threw to the participant (bad); and one threw equally to the participant and the control (neutral). The control character played the same in each version of the game, always throwing equally to the participant and whichever other character was displayed on the screen. We then assessed subjective perceptions of character trustworthiness with seven-point Likert scales (1 = not at all, 7 = very) after Cyberball. Facial stimuli for the three characters with whom participants would play the trust game were counterbalanced across participants.

Participants next played the trust game while undergoing fMRI. We employed a modified, iterated trust game adapted from a previous study in our laboratory (Delgado et al., 2005a). On each trial of the task (see Figure 1B), participants played in the role of investor with one of the three partners (trustees) they met in Cyberball. Participants began every trial with $1.00 which they could choose to either keep or share with their partner. A choice to keep resulted in $1.00 going to the participant, $0.00 to the partner, and the end of that round of the trust game. A choice to share was described as an investment: the $1.00 was multiplied by a factor of 3 before being sent to the partner. Thus, the partner would receive $3.00, which could be kept (negative outcome; $0.00 for participant), or shared back evenly with the participant (positive outcome; $1.50 for participant, $1.50 for partner).

Unbeknownst to participants, all trustees were programmed to reciprocate participant trust decisions in an equivalent manner. If participants chose to share, they would receive positive and negative outcomes approximately an equivalent number of times across social and non-social conditions. We also included a set of lottery trials. On these trials, a photo of lottery balls being pulled from a basket was presented, and the word “Lottery” was presented underneath. Participants similarly began these trials with $1.00. They could choose to keep the $1.00 and not play the lottery, or they could choose to play the lottery for a chance to win $1.50. Lottery trials were included as a non-social control condition (e.g., equivalent risk level and amounts of money at stake), and outcomes were similarly predetermined. As a check we examined average reciprocation rate as a function of condition (Mean reciprocation rate = 0.48, SD = 0.11).

All trials (see Figure 1B) began with a decision phase (2 s) in which participants were presented with a picture of one of the three partners, or the lottery condition, and two boxes on the bottom of the screen reminding them of their decision options. Participants’ decisions were collected using an MRI-compatible fiber optic response box (Current Designs, Inc., Philadelphia, PA, USA). A jittered inter-stimulus interval (10–12 s) separated the decision phase from the outcome phase. During the outcome phase (2 s), participants were presented with a box depicting one of three possibilities based on their responses [“You have kept the money” – null trial, “(partner name) has chosen to keep the money” – negative trial or “(partner name) has chosen to share the money” – positive trial]. Lottery trials were similarly presented (“You have kept the money,” “You have lost the lottery,” or “You have won the lottery”). Trials were separated by a jittered intertrial interval (10–12 s). Failure to make a response resulted in presentation of the # symbol indicating a missed trial. No penalty was administered for a missed trial; however, participants were told that it was important to respond on all trials because they would be paid additional bonuses based on outcomes from randomly chosen trials in the task. The trust game consisted of 96 total trials, separated into six functional scanning runs of 16 trials each. Each condition (good, bad, neutral, lottery) was presented 24 times. Stimuli were presented using a back projection system. The trust game was programmed using E-Prime v. 2.0 (Psychology Software Tools, Pittsburgh, PA, USA).

Upon completion of the trust game, participants made post-session ratings of trustworthiness of each character on seven-point Likert scales (1 = not at all, 7 = very). We also asked participants to rate approximately how often (e.g., percentage) they thought each of the partners shared back with them during the trust game. All participants were paid both their participation rate and additional bonuses from real outcomes of randomly chosen trials.

All participants returned for a follow-up behavioral session no more than a week from the scan session. Here, participants played a second trust game with a different set of partners, whose moral character (good, bad, neutral) was portrayed via instructed learning – i.e., fictional biographical vignettes (Delgado et al., 2005a). Participants were instructed that they would be playing with three different fictional partners whom they would get to know by reading a few short stories about them. Participants read three vignettes and then rated each character on measures of trustworthiness. Participants then played a trust game with these partners as per that described on Day 1. Upon completion of the trust game, participants were again paid real outcomes from randomly chosen trials. Average total payment for participation plus all task bonuses (e.g., Day 1 + Day 2) was $67.00. All participants were fully debriefed at the end of the session as to the predetermined nature of reinforcement and the purpose of the study.

### Behavioral analysis: Subjective ratings

We assessed the effectiveness of the Cyberball manipulation to instill social impressions, as well as changes in these impressions after the trust game by entering participants’ trustworthiness ratings at both time points into a 2 (time: pre/post) × 3 (condition) repeated measures ANOVA. Ratings of likeability were submitted to the same analyses. We also compared participants’ subjective assessments of how often their trust was reciprocated during the trust game by each of the three partners using a one-way repeated measures ANOVA. *Post hoc* *t*-tests were conducted to further examine resulting significant effects. If a family of *post hoc* tests consisted of two or more comparisons, we corrected for multiple comparisons using the Sequential Bonferroni Method (Holm, 1979; Rice, 1989). The same analyses were conducted on subjective ratings from the Day 2 behavioral session.

### Behavioral analysis: Trust game behavior

We assessed participants’ decisions to share as a function of condition in the trust game using a one-way repeated measures ANOVA. Where appropriate, *post hoc* comparisons were corrected using the Sequential Bonferroni Method (Holm, 1979; Rice, 1989). Behavior from the Day 2 session was subjected to the same analyses.

### Behavioral analysis: Reinforcement learning models

To gain a greater understanding of how participants learned to trust, we employed an abstract computational model, which mathematically represents how participants make decisions. This approach has been employed to understand the computational processes underlying social decision-making (Chang et al., 2010) and has been combined with fMRI to highlight the neural processes associated with these computational processes (O’Doherty et al., 2007; Behrens et al., 2008; Hampton et al., 2008; Chang and Sanfey, 2011; Chang et al., 2011; Jones et al., 2011). We used a simple approach to model the Expected Value (EV) of a given choice, which we defined as the mathematical product of the monetary value of the choice and the probability of realizing that choice (see Eq. 3). However, as the probability of partner reciprocation in the trust game is unknown, we used a simple Rescorla–Wagner PE rule (Rescorla and Wagner, 1972) from reinforcement learning theory (Sutton and Barto, 1998) to update participants’ beliefs about the likelihood of partner reciprocation after each encounter. This algorithm is akin to temporal-difference learning (Sutton and Barto, 1998; O’Doherty et al., 2003) and updates the belief about the likelihood of reciprocation by subtracting the experienced outcome from the expected outcome (see Eq. 1). We chose to allow the beliefs to update differentially based on whether the feedback was experienced in the context of a loss or gain (LG Model; Frank et al., 2007). More formally, the perceived probability *p* of partner *i* reciprocating at time *t* can be operationalized as

(1) $$\begin{array}{c} p_{i}\left(t\right)=p_{i}\left(t-1\right)+\alpha_{\text{gain}}*\max\left(\gamma -p_{i}\left(t-1\right),0\right)+\alpha_{\text{loss}}\\ {}*\min\left(\gamma -p_{i}\left(t-1\right),0\right) \end{array}$$

where

(2) $$\text{γ}=\left\{ \begin{array}{l} 1\text{ when partner shares}\\ \;0\text{ when partner Keeps} \end{array}\right.$$

and α_gain_ and α_loss_ are constrained between 0 and 1. The starting beliefs for all conditions *p_i_*(0) were initialized to 0.5 for maximum uncertainty. The EV of partner *i* at time *t* is calculated by multiplying the participant’s perceived probability of their partner reciprocating *p_i_*(*t*) by the amount of money they will receive if their partner reciprocates, which is half of the participant’s tripled $1 investment (i.e., $1.5).

(3) $$\begin{array}{c} \text{E}{\text{V}}_{i}\left(t\right)=p_{i}\left(t\right)*v_{i}\left(t\right) \end{array}$$

The probability *Pr* of a participant investing over keeping their money for partner *i* at time *t* was determined by placing the EV*_i_*(*t*) into a softmax function.

(4) $$\begin{array}{c} pr_{i}\left(t\right)=\frac{e^{\frac{\text{E}{\text{V}}_{i}\left(t\right)}{\beta }}}{e^{\frac{EV_{i}\left(t\right)}{\beta }}+e^{\frac{1}{\beta }}} \end{array}$$

where 0 ≤ β ≥ 1 and reflects the temperature of how much the participant explores or exploits a strategy. The EV for keeping for all decisions was $1 with 100% certainty (i.e., 1).

The model parameters were estimated in MATLAB (Mathworks, MA, USA) using the fmincon optimization function by maximizing the log-likelihood of the data under the model on a trial-to-trial basis. One hundred randomly selected start locations using RMsearch reduced the likelihood that the model converged on a local minimum. Log-likelihood estimates (LLE) were calculated separately for each participant as

(5) $$\begin{array}{c} \text{LLE}=\sum_{t=1}^{n}\log\left(pr_{i,j}\left(t\right)\right) \end{array}$$

where *i* is the partner, *j* is the action (i.e., share or keep), *t* is the trial, and *n* is the total number of trials.

To ensure that participants were actually learning the probability of their partner’s reciprocation via PE, we ran a control model which assumed a fixed 50% probability of reciprocation and therefore no learning (NL Model). Specifically, the EV for each condition *i* at trial *t* was computed by setting *p_i_*(*t*) in Eq. 3 to 0.5 and then using the identical maximum log-likelihood procedure via Eqs 4 and 5. This model only included one free parameter for the β temperature parameter in the softmax function. We were additionally interested in demonstrating that our results could not be explained by a simple intercept model in which participants formed expectations about the probability of their partner reciprocating based on their cyberball interactions, but then did not update these beliefs. This no learning initialization model (NL Init) was formulated similarly to the NL Model, but participants’ beliefs θ about the probability of each partner *i* reciprocating at time *t* was estimated separately for each partner by

(6) $$\begin{array}{c} \text{E}{\text{V}}_{i}\left(t\right)=\theta_{i}\left(t\right)*1.5 \end{array}$$

where θ*_i_*(*t*) is a free parameter constrained between 0 and 1. This model thus had five free parameters, one for each partner type (*n* = 4) and a temperature parameter for the softmax function. As a more stringent control model, we ran an additional model which combined the initialization model with the LG learning model (LG Init). This model contained seven free parameters: two learning rates (Alpha Gain and Alpha Loss), a temperature parameter for the softmax function (beta), and four initialization parameters (one for each partner type). We then used the Akaike Information Criterion (AIC; Akaike, 1974) which penalizes models for the number of free parameters as a metric of model fit and compared model fits using paired *t*-tests.

Finally, we fit an additional model to examine if participants’ differentially learned from gains or losses as a function of their interaction partner. This model was formulated almost identically to Eq. 1 with the exception that we fit separate learning rate and temperature parameters for each partner *i*.

(7) $$\begin{array}{c} p_{i}\left(t\right)=p_{i}\left(t-1\right)+\alpha_{\text{gai}{\text{n}}_{i}}*\max\left(\gamma -p_{i}\left(t-1\right),0\right)+\alpha_{\text{los}{\text{s}}_{i}}\;\\ {}*\min\left(\gamma -p_{i}\left(t-1\right),0\right) \end{array}$$

(8) $$\begin{array}{c} pr_{i}\left(t\right)=\frac{e^{\frac{\text{E}{\text{V}}_{i}\left(t\right)}{\beta_{i}}}}{e^{\frac{\text{E}{\text{V}}_{i}\left(t\right)}{\beta_{i}}}+e^{\frac{1}{\beta_{i}}}} \end{array}$$

The parameters estimated from this model were compared using mixed effects regression from the LME4 package in the R statistical language allowing for randomly varying intercepts and slopes. We employed one-tailed hypothesis tests on these parameters as we had specific predictions about their directionality. It should be noted that these types of analyses must overcome noise compounded from two estimation procedures (i.e., the parameter estimated from the computational model and also the statistical model), thus the hypothesis tests are necessarily weaker with this increased variability.

Identical modeling analyses were conducted on trust game behavior from the Day 2 behavioral session.

### fMRI acquisition and analysis

Images were acquired using a 3T Siemens Allegra head-only scanner. Structural images were collected using a T1-weighted MPRAGE sequence (256 × 256 matrix, FOV = 256 mm; 176. 1-mm sagittal slices). Functional images were acquired using a single-shot gradient echo EPI sequence (TR = 2000 ms, TE = 25 ms, FOV = 192 cm, flip angle = 80°, bandwidth = 2604 Hz/Px, echo spacing = 44) and comprised 35 contiguous oblique-axial slices (3 mm × 3 mm × 3 mm voxels) parallel to the anterior commissure-posterior commissure (AC-PC) line. BrainVoyager QX software (Version 2.2, Brain Innovation, Maastricht, The Netherlands) was used to preprocess and analyze the imaging data. Data was preprocessed using: three-dimensional motion correction (six parameters); slice scan time correction (cubic spline interpolation); spatial smoothing, using a three-dimensional gaussian filter (8-mm FWHM); voxel-wise linear detrending; high-pass filtering of frequencies (three cycles per time course). Structural and functional data of each participant were then transformed to standard Talairach stereotaxic space (Talairach and Tournoux, 1988).

We conducted a random effects general linear model (GLM), modeling decisions and outcomes as a function of condition (good/bad/neutral/lottery). Thus, this model included a total of 27 regressors including: keep and share decision regressors for each condition type (8), positive and negative outcome regressors for each condition type (8), a keep regressor for each condition type (4) during the outcome phase (e.g., corresponding to the presentation of feedback for participants’ choices to keep and not share), and seven additional regressors of no interest (six motion parameters and one regressor indicating missed trials). To investigate outcome related BOLD activation, we conducted a 2 (outcome type: positive/negative) × 4 (condition: good/bad/neutral/lottery) whole brain repeated measures ANOVA in BrainVoyager. Similarly, to characterize BOLD activation during the decision phase, we conducted a 2 (decision type: keep/share) × 4 (condition) whole brain repeated measures ANOVA in BrainVoyager. We extracted mean parameter estimates from clusters showing significant effects in order to characterize BOLD activity during the outcome phase of the task. *Post hoc* *t*-tests were conducted to further examine significant effects, and were corrected for multiple comparisons using the Sequential Bonferroni Method (Holm, 1979; Rice, 1989).

We also conducted a parametric analysis to examine BOLD activation that correlated with PE during the outcome phase. Using trial-to-trial PE values derived from the LG learning models run on our behavioral data, we constructed two additional random effects GLMs. First, to generally characterize PE related activation, we included three regressors: two dummy coded regressors corresponding to the presentation of the decision and outcome phases, and one parametric PE regressor, collapsed across all conditions. This model allowed us to search for areas in the brain that parametrically tracked with social PE at a trial-to-trial level (Jones et al., 2011). As noted above, two participants demonstrated behavioral patterns that contained insufficient variability such that they never experienced positive outcomes with the bad character. This led to biased estimation of model parameters – e.g., attempted estimation of α_gain_ for the bad character, which would be inaccurate given the lack of occurrence of this condition. Thus, these participants were excluded from group analysis so as not to unduly bias model estimates. We constructed a second model both to explore whether PE related activity varied by condition, and to ensure that observed PE responses were not due solely to lottery trials. Here we included two dummy coded regressors corresponding to the decision and outcome phases, as well as four parametric regressors corresponding to PE values for each condition (good, bad, neutral, lottery). We note that very small PE values for some runs of certain participants led to matrix inversion errors during singular value decomposition of the design matrix in BrainVoyager. This unfortunately results in an inaccurate estimation of the GLM. As such, we were forced to exclude several participants’ runs in the second model (12 runs total; five participants had 1–3 runs excluded for this reason) in order to ensure that the model was estimated correctly. For both parametric models, seven regressors of no interest were included (six motion parameters and one regressor indicating missed trials). At the group level, we examined one-sample *t*-tests of the parametric regressor, which revealed brain areas that linearly tracked with the model-derived PEs consistently across participants.

In addition, we probed relationships between PE related activity and behavioral model parameters (learning rates) via correlations at the whole brain level. This exploratory analysis revealed areas of the brain in which BOLD activation was both correlated with our parametric PE regressor and modulated by subject specific learning rate parameters as derived from the LG reinforcement learning models. Using variability in individual differences, this multilevel moderation analysis allowed us to search for regions of the brain that were computationally responsible for adapting beliefs from PEs in the context of gains and losses (i.e., positive or negative PE). Subject specific learning rate parameters for both gains and losses were entered as second level regressors within the same model. Thus, any significant effects associated with one parameter will be statistically independent from the other. This analytic technique is similar to that employed by Behrens et al. (2008) and should provide similar results to studies that have employed dynamic learning rates (Krugel et al., 2009). To ensure the resulting significant correlations were not driven by outliers, we also used robust regression (robust package in R with MM estimator) to predict extracted PE related parameter estimates using the model estimated learning rate parameters (Chang et al., 2011).

For all imaging analyses, all regressors were *z*-transformed; regressors of interest were convolved with a two-gamma hemodynamic response function at the single participant level. All generated SPMs were thresholded at an uncorrected threshold of *p *< 0.001, except for whole brain correlations with model-derived learning rates, which were thresholded at an uncorrected threshold of *p *< 0.005 due to their more exploratory nature. We corrected for multiple comparisons at the group level using the Cluster Level Statistical Threshold Estimator plugin in BrainVoyager. This method of correction (Forman et al., 1995; Goebel et al., 2006) runs a series of Monte Carlo simulations across the whole brain to assess the probability that observed significant clusters of activity are not false positives, leading to a corrected threshold of *p *< 0.05. All maps were corrected using a cluster threshold of three contiguous voxels (equivalent to 81 mm^3^) of brain tissue as determined by the plugin, except for maps depicting whole brain correlations between model parameters and PE related activation (cluster threshold of seven contiguous voxels, 189 mm^3^). Mean parameter estimates were extracted from clusters surviving correction in all analyses using three-dimensional cluster spreads.

## Results: Day 1

### Behavioral results: Subjective ratings

**Figure 2:**
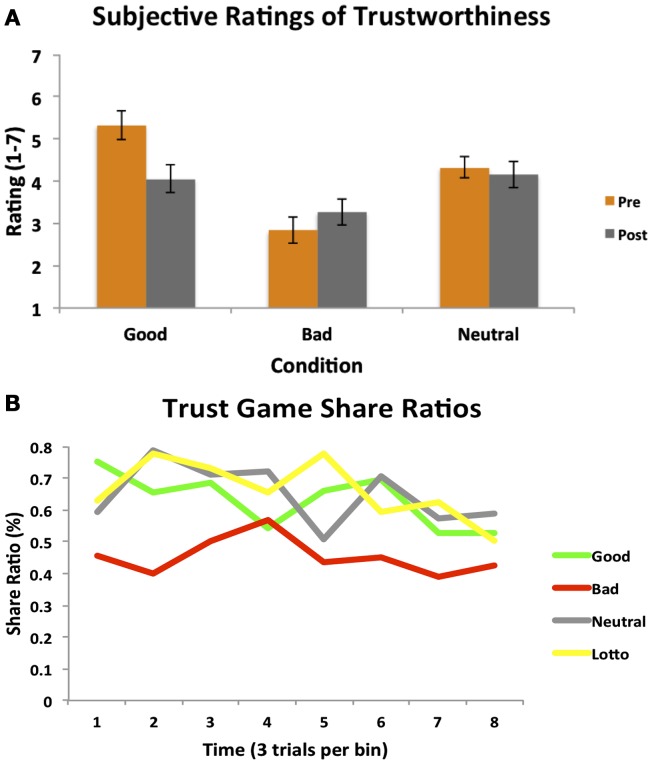
**Subjective ratings and trust game behavior**. **(A)** A 2 (time: pre/post) × 3 (condition) repeated measures ANOVA on subjective ratings of partner trustworthiness before and after the Trust Game revealed both a main effect of condition, as well as a significant time × condition interaction. **(B)** A one-way repeated measures ANOVA on participants’ trust decisions indicated a significant main effect of condition. Participants chose to share significantly less with the bad partner as compared to all other partners and the amount of time they decided to play the lottery. Participant behavior is plotted per condition as a function of time (24 trials per condition, binned into eight bins of three trials each for illustrative purposes).

Participants’ experience in the Cyberball game was an important aspect of our design, as we were interested in whether this would effectively instill social impressions and manipulate perceptions of trustworthiness. We collected subjective ratings of trustworthiness for each partner both after Cyberball (pre-trust game) and at the end of the fMRI session (post-trust game). A 2 (time: pre/post) × 3 (condition) repeated measures ANOVA revealed a significant main effect of condition [*F*_(1.458, 24.78)_ = 15.72, *p *< 0.001; see Figure 2A]. Participants rated the bad partner as significantly less trustworthy than the good [*t*_(17)_ = 4.35, *p* = 0.0004] and neutral [*t*_(17)_ = 4.05, *p* = 0.0008] partners. The good partner was rated as marginally more trustworthy than the neutral partner [*t*_(17)_ = 2.08, *p* = 0.05]. A significant time × condition interaction also emerged [*F*_(2, 34)_ = 6.61, *p *< 0.005]; this was driven by participants rating the good partners significantly less trustworthy at the end of the trust game compared to prior to playing the game [*t*_(17)_ = 2.44, *p* = 0.026; trend after Sequential Bonferroni Correction], and not significantly different than the neutral partner by the end of the task [*t*_(17)_ = 0.44, *p* = 0.68]. Participants thus did adjust their perceptions of character trustworthiness by the end of the experiment, demonstrating some explicit updating of their initial impressions.

We additionally assessed participants’ subjective perceptions of how often (e.g., what percentage of time) each partner shared back with them in the trust game. A one-way repeated measures ANOVA revealed a significant effect of condition [*F*_(2, 34)_ = 7.23, *p *< 0.003]. Participants believed that the bad partner reciprocated significantly less than the neutral partner [*t*_(17)_ = 3.69, *p* = 0.002], and marginally less than the good partner [*t*_(17)_ = 1.94, *p* = 0.07]. A similar marginal effect was observed for the comparison between the good and neutral partners [*t*_(17)_ = 1.94, *p* = 0.07], with participants believing the good partner reciprocated less than the neutral.

### Behavioral results: Trust game behavior

The focus of this study was to investigate whether initial social impressions fostered through experience in the Cyberball game would generalize to influence participants’ decisions to trust and affect the ability to modify behavior based on subsequent interaction outcomes. Results from a one-way repeated measures ANOVA on the proportion of time participants’ chose to share with each partner revealed a significant main effect of condition [*F*_(3, 51)_ = 7.06, *p *< 0.001]. Participants demonstrated a strong propensity to share less with the bad partner as compared to the good [*t*_(17)_ = 3.21, *p* = 0.004], and neutral partners [*t*_(17)_ = 3.56, *p* = 0.002]. Only the bad condition differed from the lottery condition [*t*_(17)_ = 3.87, *p *= 0.001]. We note that the relatively high rate of playing the lottery here suggests a propensity toward risk-taking behavior in general; this might be reflective of the low stakes nature of the lottery condition (Prelec and Lowenstein, 1991). Participants chose to share equally with the good and neutral partners [*t*_(17)_ = 0.42, *p* = 0.68], reflecting their similar subjective perceptions of these partners. Participants’ behavior is plotted as a function of time in Figure 2B. These behavioral results thus suggest that trust game behavior was influenced to a degree by participants’ initial experience.

### Behavioral results: Modeling analysis

The parameters for the computational models were estimated using maximum log-likelihood and are reported in Table 1. First, we demonstrate that participants did indeed use PE to learn the probability of their partner reciprocating their trust as the LG learning model fit the participants data significantly better than the NL model, which assumed a fixed 50% reciprocation rate [*t*_(17)_ = −4.64, *p *< 0.001]. Second, while the NL Init Model estimated beliefs about the probability of reciprocating that paralleled participants overall investment decisions [average values of initial parameters – Pos = 0.80 (0.23); Neu = 0.80 (0.25); Neg = 0.61 (0.22); Com = 0.86 (0.13)], this model also did not explain the behavioral data as well as the LG model [*t*_(17)_ = 3.25, *p *= 0.004]. Further, a combined LG Init model, which allowed for both participants’ initial beliefs to vary and also for belief updating via PE did not perform as well as the LG Model [*t*_(17)_ = 2.11, *p* = 0.05], [average values of initial parameters – Pos = 0.70 (0.20); Neu = 0.76 (0.13); Neg = 0.65 (0.13); Com = 0.70 (0.20)]. This suggests that participants did not merely use their initial beliefs based on their experience in the Cyberball game as the basis for their decisions in the Trust Game, but rather appeared to use trial-to-trial learning to update these beliefs based on their iterative experiences. In addition, we found evidence that the partner type appeared to differentially influence how participants learned from gains and losses. A mixed effects regression with randomly varying intercepts and slopes revealed that participants were more likely to update their beliefs for the good partner in the context of gains (i.e., α_gain_) compared to the neutral and bad partners [β = −0.18, SE = 0.10, *t*_(17)_ = −1.77, *p *< 0.05 and β = −0.21, SE = 0.11, *t*_(17)_ = −1.95, *p *< 0.05, respectively, one-tailed]. In contrast, participants were more likely to update their beliefs in the context of losses (i.e., α_loss_) for the bad partner compared to neutral partners [β = −0.10, SE = 0.06, *t*_(17)_ = −1.74, *p *< 0.05, one-tailed] and a non-significant trend for the bad partners compared to good partners and the lottery trials [β = −0.08, SE = 0.06, *t*_(17)_ = −1.36, *p *< 0.1, β = −0.10, SE = 0.07, *t*_(17)_ = −1.47, *p *< 0.1, respectively one-tailed]. There were no significant differences observed for the β parameters.

**Table 1:** **Estimated model parameters for cyberball manipulation**.

	Mean α_gain_ (SD)	Mean α_loss_ (SD)	Mean β (SD)	Mean AIC (SD)
NL			0.97 (0.14)	69.74 (3.07)^-^
NL – Init			0.44 (0.20)	66.18 (5.14)^-^
LG	0.60 (0.29)	0.06 (0.06)	0.45 (0.26)	63.60 (3.63)*
LG – Init	0.11 (0.18)	0.02 (0.02)	0.13 (0.19)	65.65 (5.81)^-^
LG – Good	0.68 (0.37)*	0.08 (0.09)	0.44 (0.31)	18.90 (3.08)
LG – Neutral	0.51 (0.33)^-^	0.06 (0.11)^-^	0.38 (0.30)	18.59 (2.85)
LG – Bad	0.48 (0.36)^-^	0.16 (0.26)*	0.39 (0.33)	19.33 (2.21)
LG – Lottery	0.62 (0.35)	0.07 (0.06)	0.48 (0.35)	19.13 (2.77)

NL refers to the No Learning model estimated for each subject, which assumed a reciprocation rate of 50%. NL – Init refers to the No Learning Initialization model, which estimated a parameter for each partner reflecting the probability of their reciprocation. LG refers to the overall Loss Gain reinforcement learning model estimated for each subject. LG – Init refers to an overall Loss Gain learning model that allowed for initial beliefs of partner reciprocation to vary. LG – Good/Neutral/Bad/Lottery reflect parameters from the LG model estimated separately by condition for each subject. *p < 0.05, ^-^comparison group.

### Neuroimaging results

**Figure 3:**
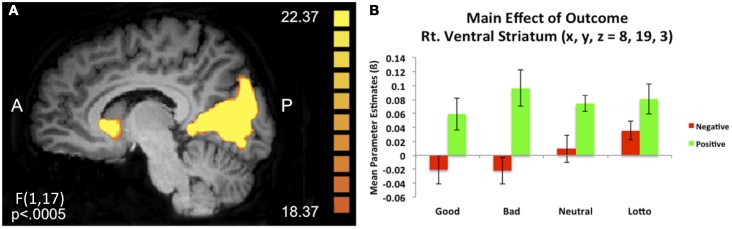
**Main effect of outcome**. **(A)** A 2 (outcome type: positive/negative) × 4 (condition) repeated measures whole brain ANOVA revealed a main effect of outcome in corticostriatal circuitry, including the right ventral caudate nucleus (*x*, *y*, *z* = 8, 19, 3). **(B)** Extracted mean parameter estimates from this ventral striatum cluster revealed increased BOLD responses to positive compared to negative outcomes, irrespective of condition.

We were additionally interested in whether initial social perceptions formed via experience may modulate outcome processing during subsequent trust interactions. An outcome × condition whole brain ANOVA during the outcome phase of the trust game revealed a number of clusters of activation demonstrating a main effect of outcome (see Table 2). Of particular interest was a cluster of activation in the right ventral caudate nucleus (*x*, *y*, *z* = 8, 19, 3) which extended slightly into the subgenual anterior cingulate cortex (sgACC). This cluster demonstrated increased BOLD responses when receiving positive compared to negative outcomes [*t*_(17)_ = 5.42, *p* = 0.00005; see Figure 3]. A cluster of activity additionally emerged in the right putamen (*x*, *y*, *z*, = 23, 4, −6) demonstrating a main effect of outcome and showed the same positive > negative response.

**Table 2:** **Regions showing main effects of outcome and condition in a 2 (outcome type) × 4 (condition) whole brain ANOVA**.

Region of activation	Brodmann area (BA)	Laterality	Talairach coordinates			# Voxels (mm^3^)	*F*-stat
			*x*	*y*	*z*	
**MAIN EFFECT OF OUTCOME**							
Inferior temporal gyrus	BA20	R	53	−17	−30	382	25.70
Inferior frontal gyrus	BA47	L	−25	28	−21	216	33.18
Inferior temporal gyrus	BA20	R	50	−35	−15	687	34.95
Fusiform gyrus	BA37	L	−46	−59	−15	586	26.27
Middle temporal gyrus	BA21	L	−52	−38	−12	383	23.77
Putamen		L	−16	1	−6	151	20.12
Putamen		R	23	4	−6	692	28.96
Lingual gyrus/cuneus	BA18/17	L	−19	−71	3	12269	68.46
Caudate nucleus		R	8	19	3	2477	40.43
Insula		L	−34	7	3	164	24.17
Cuneus	BA18	R	11	−98	6	196	23.25
Cuneus	BA17/18	R	5	−83	12	11352	59.17
Superior temporal gyrus	BA22	L	−46	−38	12	185	24.65
Inferior parietal lobule	BA7	L	−28	−59	33	228	23.46
Pre central gyrus	BA6	L	−52	−2	30	147	26.01
Posterior cingulate/precuneus	BA31	L	−4	−41	36	276	20.41
Precuneus	BA7	L	−13	−65	36	151	20.83
Inferior parietal lobule	BA40	R	41	−59	45	521	20.29
Middle frontal gyrus/precentral gyrus	BA6/4	R	23	10	54	292	22.69
**MAIN EFFECT OF CONDITION**							
Middle frontal gyrus	BA47/10	R	47	46	−3	206	9.35
Midbrain		L	−7	−11	−12	192	10.54
Cingulum		L	−13	34	15	89	8.25

We observed a main effect of condition (see Table 2) in a cluster within the midbrain (*x*, *y*, *z* = −7, −11, −12). BOLD responses here were more positive when processing outcomes from interactions with the neutral partner compared to the good [*t*_(17)_ = 6.02, *p* = 0.00001] and bad [*t*_(17)_ = 3.26, *p* = 0.005] partners. Midbrain BOLD responses were also more positive when processing outcomes with the bad as compared to the good character [*t*_(17)_ = 2.28, *p* = 0.04; trend after Sequential Bonferroni Correction]. A main effect of condition additionally emerged in middle frontal gyrus (*x*, *y*, *z* = 47, 46, −3). This cluster demonstrated increased BOLD responses during outcomes experienced after interactions with the bad partner compared to the good [*t*_(17)_ = 3.23, *p* = 0.005] and neutral [*t*_(17)_ = 2.67, *p* = 0.016] partners. No differences were observed in middle frontal gyrus BOLD responses between the good and neutral partners [*t*_(17)_ = 0.30, *p* = 0.77]. No significant interaction effects were observed between condition and outcome.

For completeness we additionally investigated BOLD responses during the decision phase of the trust game using a 2 (decision) × 4 (condition) whole brain repeated measures ANOVA. We report clusters demonstrating significant main effects of decision and condition in Table 3. However, as our focus was on outcome processing as a function of prior direct social experience, we do not discuss the decision phase results further here.

**Table 3:** **Regions showing main effects of decision and condition in a 2 (decision type) × 4 (condition) whole brain ANOVA**.

Region of activation	Brodmann area (BA)	Laterality	Talairach coordinates			# Voxels (mm^3^)	*F*-stat
			*x*	*y*	*z*	
**ME of decision**							
Occipital cortex	BA 18	R	20	−74	−6	16009	72.97
Cerebellum/fusiform gyrus	BA37	R	26	−44	−12	2401	40.94
Post central gyrus	BA1/2	R	−40	−26	57	1059	23.91
**ME of condition**		L					
Uncus	BA28/36		32	−2	−30	91	8.51
Cerebellum			26	−47	−30	100	7.41
Brainstem		R	8	−38	−30	239	8.94
Midbrain		R	5	−14	−24	390	10.90
Cerebellum		R	−19	−29	−21	292	9.34
Fusiform gyrus	BA37	R	−28	−41	−12	1144	13.36
Optic radius		L	−31	−20	−3	576	9.72
Inferior temporal gyrus	BA37	L	−58	−53	−3	665	9.25
Middle temporal gyrus	BA21	L	−61	−11	−3	630	11.94
Thalamus		L	−7	−2	3	1951	10.39
Medial occipital gyrus	BA19	L	−52	−77	6	407	8.46
Inferior frontal gyrus	BA44/45	L	−52	22	15	254	9.07
Occipital-frontal fasciculus		L	20	1	24	455	10.94
Paracentral lobule	BA7	L	8	−35	54	83	7.40

### Neuroimaging results: Modeling analysis

**Figure 4:**
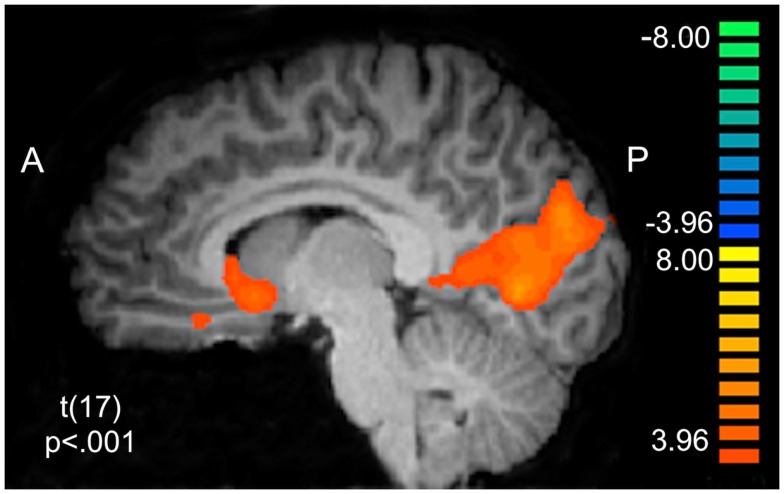
**Prediction error BOLD responses**. Model-derived trial-to-trial prediction error (PE) values were entered into a random effects General Linear Model in BrainVoyager as a parametric regressor. BOLD responses correlating with the PE regressor are observed in corticostriatal circuitry, including ventral striatum (*x*, *y*, *z* = 5, 13, −3) and ventral anterior cingulate cortex (*x*, *y*, *z* = 8, 31, −9).

We sought to characterize whether BOLD activity in putative reward circuitry was reflecting PE signals that were being used to update behavior at a trial-to-trial level. Using the PE values generated from the LG models described above as a parametric regressor, we note significant clusters of activation in Table 4. Of particular interest were clusters in right and ventral striatum extending slightly into sgACC (*x*, *y*, *z* = 5, 13, −3; see Figure 4), left ventral putamen (*x*, *y*, *z* = −16, 1, −6), and ventral ACC (BA32: *x*, *y*, *z* = 8, 31, −9). To ensure that this effect was not driven by lottery trials, we conducted an additional analysis in which only PE values for the social conditions were included (see Table 5).

**Table 4:** **Regions correlating with prediction error signals (all conditions)**.

Region of activation	Brodmann area (BA)	Laterality	Talairach coordinates			# Voxels (mm^3^)	*F*-stat
			*x*	*y*	*z*	
Inferior temporal gyrus	BA37	R	56	−56	−15	275	4.36
Anterior cingulate cortex	BA32	R	8	31	−9	173	4.43
Putamen		L	−16	1	−6	643	5.12
Ventral striatum		R	5	13	−3	3150	5.24
Cuneus/lingual gyrus	BA17/18	L	−10	−77	15	6835	6.75
Cuneus	BA18	R/L	2	−86	12	5659	6.82

**Table 5:** **Regions correlating with prediction error signals (social conditions only)**.

Region of activation	Brodmann area (BA)	Laterality	Talairach coordinates			# Voxels (mm^3^)	*F*-stat
			*x*	*y*	*z*	
Cerebellum		L	−22	−65	−18	424	5.54
Inferior temporal gyrus	BA20	R	53	−35	−15	918	7.64
Fusiform gyrus	BA18	R	26	−89	−15	263	5.41
Basal forebrain		R	8	1	−12	463	5.27
Putamen		L	−16	1	−6	672	5.26
Insula		L	−37	−11	0	113	4.73
Caudate nucleus		R	5	16	0	1403	5.30
Putamen/white matter		L	−22	19	3	1447	6.00
Middle occipital gyrus	BA19	L	−46	−80	12	195	5.21
Middle temporal gyrus	BA39	R	47	−68	15	301	4.98
Posterior cingulate/cuneus	BA31	R	5	−65	15	4520	8.64
Posterior cingulate/cuneus	BA31	L	−7	−68	15	5169	8.24
Superior occipital gyrus	BA19	L	−37	−83	30	168	5.08
Inferior parietal lobule	BA40	L	−58	−41	42	597	4.79
Middle frontal gyrus	BA6/8	L	−28	4	48	283	4.69

These results confirm the role of the ventral striatum in processing PE computations in the context of social learning (Jones et al., 2011) consistent with previously reported PE findings in non-social domains (O’Doherty et al., 2003, 2004; Daw et al., 2011). We further probed PE activation as a function of condition in these corticostriatal regions by entering extracted parameter estimates into one-way repeated measures ANOVAs. No significant effects emerged in the ventral striatum/sgACC, left ventral putamen, or ventral ACC.

### Neuroimaging results: Modulation of prediction error bold by model-derived learning rates

**Figure 5:**
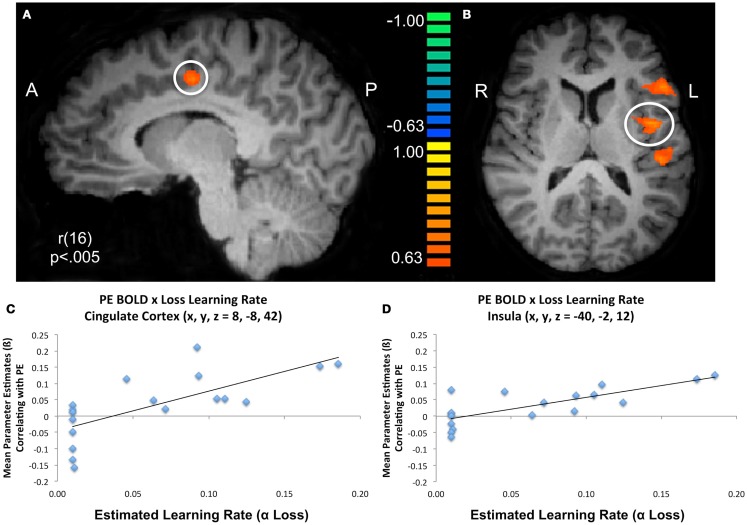
**Modulation of prediction error BOLD responses by subject specific learning rates**. Subject specific learning rates for losses (α_loss_) significantly modulated BOLD responses at the time of experienced prediction errors during the trust game in regions including **(A)** cingulate cortex, **(B)** inferior frontal/precentral gyrus, insula, and superior temporal gyrus. Scatter plots for illustrative purposes depict the relationship between the mean parameter estimates of BOLD activation that parametrically varies with model estimated prediction error values and model estimated learning rates for losses in the **(C)** cingulate and **(D)** insula.

To further characterize how participants updated their beliefs, we conducted an exploratory analysis in which we searched for areas of the brain responsible for adapting beliefs based on PE in the context of gains or losses. Specifically, we looked for neural regions which were positively associated with the parametric PE regressor and were further moderated by individual variability in participants’ learning rates as estimated by the model fitting procedure. Larger learning rate values indicate a greater weighting of PEs (i.e., when outcomes differ from expectations) when participants are updating their beliefs. In other words, PEs will lead to larger changes in trial-to-trial beliefs when learning rates are high and will result in lower changes in learning when learning rates are low. Thus, any regions that emerged in this analysis would likely be computationally involved in dynamically adapting beliefs based on positive or negative outcomes. Overall, participants demonstrated a behavioral bias toward updating behavior more readily from gains than losses. We conducted whole brain correlations between participant specific learning rates for gains and losses and BOLD activation corresponding to the PE regressor. We note that this regressor contained both positive and negative PE values. Significant positive correlations were observed between PE related BOLD responses and the α_loss_ learning parameter in regions denoted in Table 6 and illustrated in Figure 5, including two portions of inferior frontal gyrus (BA47: *x*, *y*, *z* = −37, 34, 0; BA45: *x*, *y*, *z* = −49, 19, 9), as well the insula (*x*, *y*, *z* = −40, −2, 12) superior temporal gyrus (BA22/42: *x*, *y*, *z* = −46, −26, 9) and cingulate cortex (BA24: *x*, *y*, *z* = 8, −8, 42).

**Table 6:** **Regions active in a whole brain correlation between prediction error BOLD responses and learning rates for losses (α_loss_)**.

Region of activation	Brodmann area (BA)	Laterality	Talairach coordinates			# Voxels (mm^3^)	*r*-value
			*x*	*y*	*z*	
Inferior temporal gyrus	BA20	L	−52	−32	−15	1076	0.83
Brainstem/midbrain		L	−1	−17	−3	361	0.76
Inferior frontal gyrus	BA47	L	−37	34	0	2008	0.84
Inferior frontal gyrus/precentral gyrus	BA44	L	−49	19	9	1170	0.79
Superior temporal gyrus	BA22/42	L	−46	−26	9	446	0.77
Insula		L	−40	−2	12	839	0.83
Cingulate gyrus	BA24	R	8	−8	42	752	0.76

We also observed a significant correlation between PE related BOLD responses and parameters for α_gain_ in a region bordering superior/medial frontal gyrus (BA10: 11, 67, 6). These results suggest that at the time of experienced PEs, BOLD activation in these regions was moderated by the extent to which participants used losses and gains respectively to update their beliefs. To ensure that outliers were not driving these effects, we performed a robust regression using parameter estimates for PE related BOLD activity extracted from the clusters identified here and model estimated learning rates. The results for the correlations with α_loss_ remained significant; the results for the correlation with α_gain_ were reduced to a trend.

## Results: Day 2

### Behavioral results: Subjective ratings

Similar to the results from Day 1, a 2 (time) × 3 (condition) repeated measures ANOVA using ratings both before and after the trust game revealed a significant main effect of condition [*F*_(2, 34)_ = 65.04, *p *< 0.001] such that participants rated the bad partner as significantly less trustworthy than both the good [*t*_(17)_ = 8.04, *p *< 0.001] and neutral [*t*_(17)_ = 6.28, *p *< 0.001]. A significant time × condition interaction [*F*_(1.33, 22.60)_ = 8.82, *p *< 0.005] also emerged such that participants’ ratings of the good [*t*_(17)_ = 3.06, *p* = 0.007] and bad [*t*_(17)_ = 2.70, *p* = 0.015] partners changed significantly, demonstrating explicit awareness of the notion that character behavior in the trust game was not in line with initial perceptions, replicating previous work (Delgado et al., 2005a). An additional one-way repeated measures ANOVA on participants’ subjective perceptions of partner reciprocation rates in the trust game revealed no overall effect of condition [*F*_(2, 34)_ = 1.69, *p *< 0.3].

### Behavioral results: Trust game behavior

**Figure 6:**
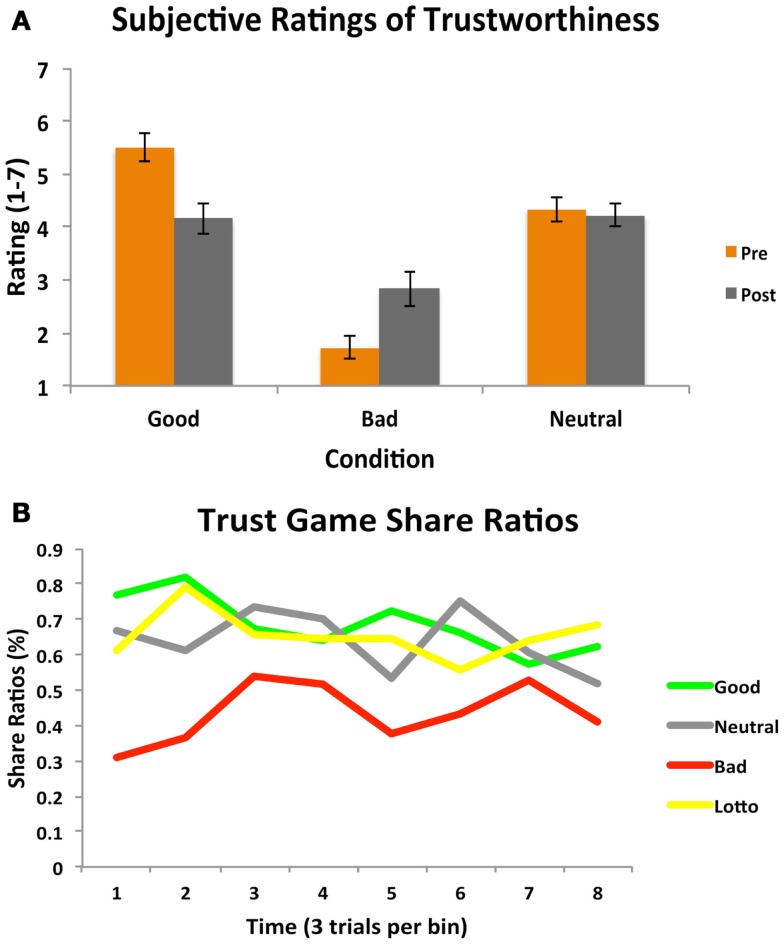
**Subjective ratings and trust game behavior (Day 2)**. **(A)** A 2 (time: pre/post) × 3 (condition) repeated measures ANOVA on subjective ratings of partner trustworthiness before and after the Trust Game paralleled results from the Day 1 session: a main effect of condition and a significant time × condition interaction emerged. **(B)** A one-way repeated measures ANOVA on participants’ trust decisions also mirrored results from the Day 1 session, showing a significant main effect of condition. Participants chose to share significantly less with the bad partner as compared to all other partners and the amount of time they decided to play the lottery. Participant behavior is plotted per condition as a function of time (24 trials per condition, binned into eight bins of three trials each for illustrative purposes).

Participants’ decisions to share or play the lottery during the follow-up trust game were entered into a one-way repeated measures ANOVA. Results (see Figure 6) indicated a significant effect of condition [*F*_(3, 51)_ = 9.34, *p *< 0.001]. In line with results from Day 1, this effect was driven by participants choosing to share less often with the bad character as compared to the good [*t*_(17)_ = 4.22, *p* < 0.001] or neutral [*t*_(17)_ = 3.97, *p* = 0.009] partners. No differences were observed between choices to share with the good and neutral conditions, however [*t*_(17)_ = 1.39, *p* = 0.18], and again, only the bad condition differed from the lottery condition [*t*_(17)_ = 3.67, *p* = 0.002].

### Behavioral results: Modeling analysis

The parameters for the computational models were again estimated using maximum log-likelihood and can be found in Table 7. Overall, the modeling results for the instructed learning paradigm were highly similar to those of the Cyberball manipulation. The LG model was a better account of the behavioral data then both the NL and the NL Init models [*t*_(17)_ = −4.46, *p *< 0.001], [*t*_(17)_ = −3.15, *p *= 0.006] respectively, and also the LG Init model [*t*_(17)_ = 2.22, *p* = 0.04]. These results suggest that participants indeed learned from PE and that their behavior cannot be simply explained by their initial expectations. Participants estimated beliefs followed their overall investment ratios for both the NL Init model [Pos = 0.88 (0.13), Neu = 0.82 (0.16), Neg = 0.58 (0.23), Com = 0.84 (0.24)] and also the LG Init model [Pos = 0.77 (0.13), Neu = 0.64 (0.17), Neg = 0.65 (0.19), Com = 0.77 (0.12)]. A mixed effects regression with randomly varying intercepts and slopes revealed that participants were more likely to update their beliefs for the good partner in the context of gains (i.e., α_gain_) compared to the bad partners and lottery conditions [β = −0.39, SE = 0.09, *t*_(17)_ = −4.36, *p *< 0.05 and β = −0.19, SE = 0.10, *t*_(17)_ = −1.87, *p *< 0.05, respectively, one-tailed]. An additional model revealed that participants were more likely to update their beliefs in the context of losses (i.e., α_loss_) for the bad partner compared to the good and neutral partners [β = −0.11, SE = 0.06, *t*_(17)_ = −1.79, *p *< 0.05, β = −0.12, SE = 0.06, *t*_(17)_ = −2.14, *p *< 0.05 respectively, one-tailed]. Participants were more exploratory in their decisions (i.e., β parameter) in the context of the neutral partner compared to the good and bad partners and lottery conditions [β = −2.68, SE = 1.53, *t*_(17)_ = −1.75, *p *< 0.05, β = −3.70, SE = 1.76, *t*_(17)_ = −2.10, *p *< 0.05, β = −2.60, SE = 1.45, *t*_(17)_ = −1.79, *p *< 0.05 respectively, one-tailed].

**Table 7:** **Estimated model parameters for instructed learning manipulation**.

	Mean α_gain_ (SD)	Mean α_loss_ (SD)	Mean β (SD)	Mean AIC (SD)
NL			1.00 (0.00)	70.96 (1.79)^-^
NL – Init			0.42 (0.12)	65.70 (6.05)^-^
LG	0.50 (0.30)	0.08 (0.11)	0.43 (0.22)	63.04 (6.00)*
LG – Init	0.16 (0.24)	0.04 (0.04)	0.12 (0.12)	65.52 (6.00)^-^
LG – Good	0.83 (0.23)*	0.08 (0.11)	0.39 (0.26)^-^	18.79 (2.66)
LG – Neutral	0.71 (0.35)	0.07 (0.10)^-^	0.54 (0.29)*	20.14 (2.16)
LG – Bad	0.44(0.35)^-^	0.19 (0.23)*	0.28 (0.20)^-^	18.29 (2.70)
LG – Lottery	0.64 (0.34)^-^	0.10 (0.19)	0.39 (0.26)^-^	18.84 (2.88)

NL refers to the No Learning model estimated for each subject, which assumed a reciprocation rate of 50%. NL – Init refers to the No Learning Initialization model, which estimated a parameter for each partner reflecting the probability of their reciprocation. LG refers to the overall Loss Gain reinforcement learning model estimated for each subject. LG – Init refers to an overall Loss Gain learning model that allowed for initial beliefs of partner reciprocation to vary LG – Good/Neutral/Bad/Lottery reflect parameters from the LG model estimated separately by condition for each subject. *p < 0.05, ^-^comparison group.

## Discussion

In the current study, we examined the neuro-computational mechanisms underlying how initial social impressions can influence subsequent learning experiences in a different domain. Our results suggest that social impressions of others acquired through direct social experience can bias subsequent learning and decision-making, particularly when that experience is negative. Consistent with extant work, we found that processing social outcomes recruits corticostriatal reward circuitry. In addition, learning from social outcomes appears to utilize the same PE driven learning system located in the ventral striatum implicated in simpler non-social associative learning tasks. Interestingly, initial impressions appear to bias how people learn from feedback in social contexts, such that learning is facilitated when feedback is consistent with initial impressions (i.e., learning from losses when playing with a bad partner and learning from gains when playing with a good partner). These results demonstrate how a decision neuroscience framework can be employed to understand the dynamics of social learning.

Forming social impressions through direct social experience effectively instilled perceptions of others. Participants differentially rated their partners according to their perceived personalities, which were effective in guiding subsequent trust game behavior; this was most robust with the bad partner, as participants chose to invest much less often in this condition. It is possible that the initial negative experience in Cyberball with the bad partner was perceived as more salient potentially due to a sense of feeling excluded. Cyberball has often been used in investigations of social exclusion (Williams et al., 2000; Eisenberger et al., 2003). While the good partner was rated as more trustworthy than the neutral, participants may have felt included by both partners in Cyberball, which may contribute to similar patterns of decision-making in the trust game. An alternative interpretation here is that the neutral partner may have been perceived as the most fair in Cyberball, due to throwing equally to the participant and the control character on the screen. If the neutral partner was indeed perceived as most fair, this may underlie the equivalent overall pattern of trust decisions with the good and neutral partners in the trust game. Nevertheless, our behavioral results are consistent with the notion that perceptions of trustworthiness provide important social signals (Adolphs et al., 1998; Willis and Todorov, 2006; Engell et al., 2007; Todorov et al., 2008) which may help guide social decision-making. While evidence exists demonstrating that decisions to trust can rely on implicit biases based on assumptions of racial group (Stanley et al., 2011), and instructed knowledge about moral aptitude (Delgado et al., 2005a), we extend these findings to show that learning about others through direct social experience can similarly influence trust behavior.

### Social outcomes recruit reward circuitry

Consistent with previous work, corticostriatal reward circuitry appears to be sensitive to social outcomes. A main effect of outcome demonstrated increased BOLD responses in regions including the striatum and cingulate cortex during positive compared to negative outcomes across social and non-social (lottery) conditions. A burgeoning body of work implicates corticostriatal reward circuitry not only in coding the value of primary (e.g., juice) and secondary (e.g., money) rewards (Delgado et al., 2000, 2004; Knutson et al., 2001, 2003; O’Doherty et al., 2002; Elliott et al., 2004; Zink et al., 2004) but also in assigning value to social rewards (for reviews see Leotti and Delgado, 2011; Rilling and Sanfey, 2011), such as approval (Izuma et al., 2010), acceptance (Somerville et al., 2006), another’s success (Mobbs et al., 2009), and shared rewards experienced within a social context (Fareri et al., 2012). Evidence also supports a role for corticostriatal reward circuitry as critical for maintaining representations of social interaction partners and their reputation (Delgado et al., 2005a; King-Casas et al., 2005; Tomlin et al., 2006; Baumgartner et al., 2008; Chang and Sanfey, 2009; Phan et al., 2010). It is interesting to note that our results somewhat diverge from previous work from our group (Delgado et al., 2005a), which demonstrated that the striatal BOLD response to positive and negative trust game outcomes with a morally reputable, or “good,” partner was blunted compared to when interacting with a neutral partner (e.g., no prior knowledge of reputation). In conjunction with behavioral results indicating behavioral biases that did not shift over the course of the experiment, Delgado et al. (2005a) interpreted their results as instructed knowledge of moral character modulating the ability of the striatum to typically process positive and negative outcomes (Delgado et al., 2000, 2005b; O’Doherty et al., 2002; Zink et al., 2004). We did not observe this blunted striatal differentiation between positive and negative outcomes as a function of partner. An ROI analysis using a 10 voxel spread around the peak striatal coordinates reported by Delgado et al. (2005a) confirmed the divergence between the two studies. Conducting a 2 (condition) × 2 (outcome) repeated measures ANOVA on extracted parameter estimates when collapsing across the “good” and “bad” conditions as per Delgado et al. (2005a) revealed no significant interaction [*F*_(1, 17)_ = 0.937, *p* > 0.3]. It is important to note that the differences in methods of initial learning between the present study and the study by Delgado et al. (2005a) may underlie the divergence in results. Nonetheless, the behavioral and neuroimaging results from the experience condition in the present study suggest that participants here may have been using trust game outcomes to continually form their impressions of their partners.

### Social learning via prediction error

Vital to assessing another as having a trustworthy reputation is the notion of reciprocity – e.g., will generous behavior be reciprocated (van den Bos et al., 2009). Previous work has indicated that people may treat partner reciprocity as a conditional probability and appear to learn the trustworthiness of a partner through PE driven learning (Chang et al., 2010). Consistent with this notion, we observed evidence that participants can successfully learn the likelihoods of their partners’ reciprocation rates over time from trial-to-trial feedback via a reinforcement learning process. Participants appeared to have an expectation about the likelihood of a partner responding and then updated these beliefs following trust game outcomes using PEs. Similar to non-social PE driven associative learning (O’Doherty et al., 2003, 2004; Pessiglione et al., 2006; Schonberg et al., 2007; Daw et al., 2011; Li et al., 2011b), the computational process of social PE appears to recruit the ventral striatum in our study. The observed social PE signals in ventral striatum are consistent with a handful of studies (Hampton and O’Doherty, 2007; Jones et al., 2011), but diverge from other work demonstrating social PE to be processed in the STS/TPJ (Behrens et al., 2008), more putatively implicated in social processes such as considering intentions of others (Amodio and Frith, 2006; Saxe, 2006). However, all of these studies have employed different tasks, which may account for these discrepancies.

It is also of interest to note that investigations of trustworthiness often report involvement of the amygdala; evidence suggests that this region is important for subserving initial social appraisals of others based on facial characteristics (Adolphs et al., 1998; Willis and Todorov, 2006; Engell et al., 2007; Oosterhof and Todorov, 2008; Todorov et al., 2008). The implication here is that the amygdala may be coding a social approach/avoid signal. This is consistent with both animal and human findings indicating that the amygdala is important for both affective valuation in general (Everitt et al., 1991, 1999; Gottfried et al., 2003; Paton et al., 2006; Belova et al., 2008), as well as animal models implicating this region in social processes (Maaswinkel et al., 1996; Ferguson et al., 2001; Markram et al., 2008; Insel, 2010). We did not observe increases in amygdala BOLD responses in our task that were specifically sensitive to social partner at the time of decision or outcome. The lack of amygdala involvement in our trust game task may be accounted for by the fact that initial impressions/evaluations of trust game partners were formed prior to the trust game. As such the trust game experience was used to continually learn reputation, which evidence suggests is akin to a striatal-based reinforcement learning process (King-Casas et al., 2005; Phan et al., 2010; Kishida and Montague, 2012). Additionally, recent findings indicate that the amygdala and striatum may perform separable contributions to (Delgado et al., 2008) learning, with the striatum supporting trial-to-trial updating of action and outcome values via reinforcement learning, whereas the amygdala may play a more prominent role in coding surprise and associability of stimuli (Li et al., 2011b).

### Evidence for confirmation bias

Participants’ initial impressions influenced how they utilized experienced social PEs to update their behavior, as reflected in participants’ estimated learning rates. Participants learned better from positive outcomes with the good partner compared to the bad partner, whereas the converse was true for losses, with higher learning rates observed for losses with the bad partner than with the good or neutral partners. This tendency to rely on information confirming initial social experience to guide behavior and inform reputation building is consistent with the notion of confirmation bias. Previous work suggests that trustworthiness perceptions may evolve dynamically, accounting for the interaction of implicit judgments of unknown others and social interaction outcomes (Chang et al., 2010), and that instructed information in non-social learning situations biases the ability to incorporate information inconsistent with said biases (Doll et al., 2009; Li et al., 2011a). While our study differs in terms of task structure and the models applied from others investigating social and non-social learning, the present results support these previous findings in that impressions formed from direct social experience may continually shape learning in a new social situation by using information that was consistent with previous biases.

### Detection of salient outcomes

Previous work on confirmation bias in the context of instructed learning demonstrates that executive control regions such as the dorsolateral prefrontal cortex (DLPFC) may provide top down input to override striatal learning weights (Li et al., 2011a). This is consistent with proposed neural network models (Doll et al., 2009) and recent work suggesting that genetic variability in the COMT gene, which provides dopamine to prefrontal regions, may account for individual susceptibility to the confirmation bias effect (Doll et al., 2011). Our results do not support the idea that participants treated Cyberball as an instructed learning task, but do suggest that social impressions influence subsequent learning to be more sensitive to feedback consistent with these impressions. Interestingly, in our exploratory imaging analyses, we find that participants’ learning rates for learning from losses significantly correlated with BOLD responses at the time of experienced PEs in regions (e.g., cingulate cortex and insula) that are involved in salience detection, monitoring unexpected outcomes, cognitive control, and switching between default and executive control networks (Bush et al., 2000; Miller and Cohen, 2001; Sridharan et al., 2008; Menon and Uddin, 2010). We do note that previous work reports activation in more rostral and anterior areas of cingulate and insular cortices than what is reported in the present study. Within this context, however, our experimental manipulation may have biased participants to be more vigilant when updating from negative outcomes, which may have been perceived as more salient. This is consistent with previous arguments that variability in learning rates may recruit the cingulate to detect outcome volatility in the learning environment (Behrens et al., 2007, 2008) and following reversal learning (Krugel et al., 2009). Previous work investigating social bargaining in the Ultimatum game implicates the anterior cingulate as critical in monitoring expectancy violations (Chang et al., 2012) which not only bias behavior, but can also result in memory enhancement for partners that violate expectations (Chang and Sanfey, 2009). While we were unable to specifically examine learning rates in the context of positive and negative PEs for each condition due to an insufficient number of trials, the combination of our modeling and imaging results suggest a possibility that initial negative impressions may predispose the brain’s monitoring system to be more sensitive to outcomes consistent with these impressions. This phenomenon may be more fruitfully explored in future work.

### Differences between instructed and experienced initial impressions

We attempted to compare initial impressions formed through direct experience and instructed means, similar to a recent investigation in the non-social domain (Li et al., 2011a). We found that initial impressions formed from instructed learning support our results from the experience learning sessions. Participants shared less with the bad partner compared to good and neutral partners, but no differences were observed between the proportion of share decisions with the good and neutral partners. Instructed learning here seemed to instill similar biases regarding learning rates, as participants were most sensitive to information consistent with initial impressions: we observed higher learning rates for positive outcomes with the good partner compared to others, and higher learning rates for negative outcomes with the bad partner compared to others. Though learning rates for the good (α_gain_ = 0.83) and bad (α_loss_ = 0.19) partners appeared stronger than those observed during the experience learning session on Day 1 for the good (α_gain_ = 0.68) and bad (α_loss_ = 0.16) partners, the LG model did not fit the experience or instructed learning sessions differently [*t*_(17)_ = 0.12, *p* = 0.91], nor were there overall differences in comparing overall learning rates across the two sessions α_gain_: [*t*_(17)_ = −0.62, *p* = 0.91]; α_loss_: [*t*_(17)_ = 1.12, *p* = 0.28]}. The results for the instructed condition here do differ slightly from the experience learning condition in that participants were more exploratory in their decisions with the neutral partner compared to all others reflected in their estimated β parameter; this pattern was absent after forming impressions from experience, suggesting a difference in the manner in which the neutral partner was perceived across the two types of manipulations. As noted above, it is possible that the neutral Cyberball partner may have been perceived as more fair; participants may have thus formed a more positive impression of this partner after Cyberball compared to the more uncertain information associated with the neutral biographical vignette in the instructed condition.

We do note an important point when comparing the two sessions in the present study. The follow-up instructed learning session took place up to 1 week after the imaging session. The trust game paradigms were identical, save for the partners, names, and method of acquiring impressions. It is possible that due to prior experience with the trust game, practice, and order effects may have influenced the follow-up results. Participants may have relied in some way on this prior experience with the paradigm to guide behavior here rather than, or in conjunction with, the initial impressions formed from instructed learning. While the learning rate comparisons do seem to be in the same direction as previous findings showing enhanced performance when learning via instructed means (Li et al., 2011a), it is difficult to make this claim without a significant comparison in our task. Future work investigating differences in updating partner reputation after initial experience or instructed means could better control for these potential confounds by conducting both tasks within the same imaging session and controlling for order.

## Conclusion

Assessing whether another person is trustworthy and subsequently learning their reputation is critical to survival in a social world. This lays the foundation for various types of relationships. It is likely that we will often use initial social knowledge to inform subsequent behavior, whether it be from implicit or assumed biases, instructed third party information, or prior direct experience. Here, we demonstrated that social impressions formed from direct social experience could generalize to a different domain, biasing decisions to trust and priming sensitivity to subsequent social outcomes that confirm these impressions, particularly within a negative social context.

## Conflict of Interest Statement

The authors declare that the research was conducted in the absence of any commercial or financial relationships that could be construed as a potential conflict of interest.
